# Identification of cannabinoid-sensitive and -resistant oral bacteria

**DOI:** 10.3389/fmicb.2025.1709243

**Published:** 2026-01-05

**Authors:** David A. Scott, Gwyneth J. Lamont, Jinlian Tan, Arjun P. Patel, Jack T. Guffey, Scott C. Thomas, Fangxi Xu, Gill Diamond, Deepak Saxena

**Affiliations:** 1 Oral Immunology and Infectious Diseases, University of Louisville School of Dentistry, Louisville, KY, United States; 2 Molecular Pathobiology, New York University College of Dentistry, New York, NY, United States

**Keywords:** anti-microbials, cannabis, periodontitis, *Porphyromonas gingivalis*, spirochetes

## Abstract

Marijuana, an emerging risk factor for periodontitis, contains multiple potent antibacterials, particularly the phytocannabinoids. Microbial dysbiosis is a hallmark of this destructive oral disease. We examined a panel of oral bacteria for susceptibility to the major cannabinoid, cannabidiol (CBD), portended by an initial *in vivo* microbiome analysis of marijuana users and non-users with periodontitis. Multiple oral bacteria were found to be sensitive to physiologically relevant CBD doses–*Aggregatibacter actinomycetemcomitans*, *Fusobacterium nucleatum*, several strains of *Porphyromonas gingivalis*, *Streptococcus mutans*, *Streptococcus gordonii* and *Tannerella forsythia*. Other oral bacteria, however, were resistant to even superphysiological CBD concentrations–*Campylobacter gracilis, Corynebacterium durum, Haemophilus parainfluenzae*, several oral *Treponema* species and *Veillonella parvula*. Enrichment of phytocannabinoid resistant bacterial pathobionts may help explain increased periodontitis prevalence in cannabis users who, like tobacco smokers, may have distinct therapeutic and preventive needs. To this end, a library of membrane permeabilizing peptoids (*N*-substituted glycine oligomers), based on an endogenous mammalian antimicrobial peptide, cathelicidin, was screened for activity against *Treponema denticola*. This spirochete was sensitive to a sub-set of stable and inexpensive antimicrobial peptoids that, presumably due to peptoid-induced outer membrane instability, also rendered CBD toxic to normally resistant spirochetes. The tobacco-stable, cannabinoid-labile pathobiont, *P. gingivalis*, was also sensitive to specific antimicrobial peptoids. Electron micrographs clearly suggest altered ultrastructure in both CBD-treated *P. gingivalis* and peptoid-exposed *T. denticola*. In summary, cannabis use may promote specific oral bacteria while suppressing others. The associated dysbiosis may help explain marijuana-exacerbated periodontitis. While more comprehensive studies of cannabis-induced microbial fluxes are warranted, adjunctive antimicrobial agents, such as cathelicidin-mimicking peptoids, that target cannabis-promoted pathobionts may also be worth exploring for therapeutic potential.

## Introduction

Cannabis use is emerging as a major risk factor for periodontitis, with disease onset reported to occur at a younger age than is usually seen in the general population (Thomson et al., 2008; Zeng et al., 2014; Thomson et al., 2013; Jamieson et al., 2010; Hujoel, 2008; Duane, 2014; Shariff et al., 2016; Meier et al., 2016; Chaffee, 2021; Perez-Rivoir et al., 2021; Mayol et al., 2021; Javed et al., 2020; Scott et al., 2022; Panee et al., 2024). Thus, the increasing prominence of cannabis consumption, in line with reduced legislative barriers to use, may represent a growing oral health problem. However, the underlying causes of cannabis-induced and/or -exacerbated periodontitis are, essentially, unknown.

As marijuana contains multiple antimicrobials, in particular the major phytocannabinoids [cannabidiol (CBD), tetrahydrocannabinol (THC) and cannabinol (CBN)] (Tan et al., 2024; Coelho et al., 2025; Hong et al., 2022), one key predisposing mechanism may be the induction of a disease-related dysbiotic plaque. Indeed, we have earlier reported that the best studied oral pathobiont, *Porphyromonas gingivalis*, which thrives in tobacco-rich environments (Pushalkar et al., 2020; Hutcherson et al., 2020; Shah et al., 2017; Zeller et al., 2014; Zambon et al., 1996) is highly susceptible to killing by phytocannabinoids (Gu et al., 2019). *Treponema denticola* and *Treponema putidum*, two understudied periodontitis-associated oral spirochetes, on the other hand, are entirely resistant to physiologically relevant concentrations of phytocannabinoids (Gu et al., 2019; Tan et al., 2024).

Therefore, we set out to examine microbial signatures in a convenience sample of saliva specimens from marijuana users and non-users with periodontitis and to extrapolate the findings into a laboratory setting.

As cannabis-promoted pathobionts may be of particular importance in marijuana-associated disease, we also screened a library of membrane permeabilizing anti-microbial peptoids (sequence-specific *N*-substituted glycine oligomers), based on a well-characterized endogenous mammalian antimicrobial peptide, cathelicidin (LL-37), for activity against a model cannabinoid-resistant oral microbe, *T. denticola*. We also examined peptoid efficacies against a model cannabinoid-sensitive, nicotine-resistant periodontal pathogen, *P. gingivalis*. Such peptoids have been shown by us and others to exhibit membrane permeabilizing activity against bacteria, fungi and enveloped viruses (Diamond et al., 2021; Tate et al., 2023; Lin et al., 2022).

About 19% of the USA population, more than 50 million people, use cannabis (Administration, SAAMHS, 2022; CDC, 2025). The relative risk for the development of periodontitis in cannabis users has been reported to be particularly high (Thomson et al., 2008; Aljoghaiman, 2025), similar to tobacco smokers (Bergstrom, 2014; Tomar and Asma, 2000). NHANES data from subjects aged 30–59 (*n* = 1938), revealed increased cannabis-associated disease severity (PD and CAL) across all ethnicities and income brackets (Shariff et al., 2016). The most recent data, from a study population of >3,600, suggests that severe periodontitis affects 39% of cannabis users, with OR: 4.3; 95% CI: 3.7–5.0 (*p* = 0.001) compared to non-users (Aljoghaiman, 2025). The *in situ* oral microbial fluxes associated with cannabis use in subjects with periodontitis, laboratory bacterial sensitivity profiles to CBD, and the potential adjunctive efficacy of cathelicidin-mimicking peptoids are presented in this context.

## Materials and methods

### Human subject recruitment and sample collection

We previously recruited and followed longitudinal microbiome changes in a cohort of e-cigarette users and control subjects in the context of periodontal disease progression (Xu et al., 2021). A minority of the control population were non-tobacco consuming cannabis users unsuitable for the original trial but providing a convenience sample for the initial investigation of cannabis-associated bacterial fluxes. Full details of subject enrollment, recruitment and eligibility criteria; disease characteristics and classification; oral sampling; DNA extraction and sequencing; 16S rRNA gene sequence data analysis; and statistical approaches have been published (Xu et al., 2021). Briefly, periodontal measurements were recorded at six sites per tooth (mesio-buccal, buccal, disto-buccal, mesio-lingual, lingual, and disto-lingual) on all teeth present. The definition of periodontal disease was according to the CDC in collaboration with the American Academy of Periodontology (CDC-AAP) (Eke et al., 2013), where ≥ two interproximal sites with ≥3 mm attachment loss, and ≥2 mm interproximal sites with PD ≥ 4 mm (not on the same tooth), or one interproximal site with PD ≥ 5 mm was considered periodontitis. The study was approved by the Institutional Review Board of New York University Langone Medical Center. All participants provided informed written consent prior to enrollment. Participants reported their smoking status, alcohol use and dental hygiene history, completed a medical history questionnaire, and underwent a comprehensive oral and periodontal examination at enrollment. Saliva samples were collected and stored -80°C prior to further processing.

### Sequencing and analysis

DNA extractions and sequencing were performed as described previously (Xu et al., 2021; Thomas et al., 2022). Briefly, genomic DNA from samples was extracted using the MoBio Power fecal kit (MoBio Laboratories Inc., Carlsbad, CA), quantified, and checked for purity. For library preparation, the V3-V4 region of the 16S rRNA gene was amplified (10 ng/μl per sample) using the common bacterial primers 805R and 341F. Libraries were pooled in equimolar amounts, denatured, diluted, and sequenced on an Illumina MiSeq platform using the MiSeq reagent kit v3 (600 cycles) following the 2 × 300-bp paired-end sequencing protocol.

The reads were quality-checked and processed using Quantitative Insights Into Microbial Ecology v2.0 (QIIME2) (Bolyen et al., 2019). Amplicon sequence variants (ASVs) and representative sequences were generated using the DADA2 denoising algorithm (Bolyen et al., 2019; Xu et al., 2022). A phylogenetic tree was constructed with FastTree, following multiple sequence alignment using MAFFT via the qiime phylogeny align-to-tree-mafft-fasttree plugin (Callahan et al., 2016). For taxonomic classification, the HOMD database (v15.2) was used to train a Naïve Bayes classifier tailored to the 16S rRNA V3–V4 region using the primers 341F (5′-CCTACGGGNGGCWGCAG-3′) and 806R (5′-GACTACHVGGGTATCTAATCC-3′), with qiime feature-classifier extract-reads.

Downstream analyses and visualizations were performed in R (v4.0.3) using the phyloseq package (McMurdie and Holmes, 2012). To assess differences in microbial community composition between cannabis users and non-users (beta diversity), principal coordinates analysis (PCoA) was conducted using a Weighted UniFrac distance matrix. Statistical significance between sample clusters was evaluated using permutational multivariate analysis of variance (PERMANOVA) via the adonis function in the vegan package (v2.5–7). Differential relative abundance analyses were conducted using ANCOM-BC and LEfSe (Segata et al., 2011; Lin and Peddada, 2020). Sample–ASV interaction network was visualized using Cystoscope v3.8.2. Statistical significance was defined as a frd corrected *p*-value (*q*-value) < 0.05.

### Bacterial growth in the presence or absence of CBD

*Aggregatibacter actinomycetemcomitans* 652, *Campylobacter gracilis* ATCC 33236*, Corynebacterium durum* ATCC 33822, *Fusobacterium nucleatum* subsp*. nucleatum* ATCC 25586, *Haemophilus parainfluenzae* ATCC 33392, *Porphyromonas gingivalis* (ATCC 33277, 5607, 8012 and 6404), *Streptococcus gordonii* DL-1, *Streptococcus mutans* KPSP2, *Tannerella forsythia* ATCC 43037, *Treponema denticola ATCC 35405*, *Treponema putidum* ATCC 700334 and *Veillonella parvula* ATCC 10790, from our own collection or purchased from the American Type Culture Collection (Manassas, VA), were grown under the conditions provided in Supplementary Table 1. Each bacterium was cultured in the presence of multiple concentrations of CBD (0–100 μg/mL) or with the solvent control (methanol). Growth was monitored spectrophotometrically at OD_600nm_ using a Biowave CO8000 cell density meter (Biochrom Ltd., Cambridge, UK). The minimal inhibitory concentration (MIC) of CBD for each sensitive species of interest was ascertained.

### Bacterial growth in the presence or absence of antimicrobial peptoids and/or CBD

A cannabinoid-resistant bacterium, *T. denticola* ATCC 35405, and a cannabinoid-sensitive but highly tobacco-resistant bacterium (Tan et al., 2024; Hutcherson et al., 2020; Bagaitkar et al., 2011; Bagaitkar et al., 2010), *P. gingivalis* ATCC 33277, were grown in media containing serial dilutions of cathelicidin-mimicking peptoids (peptoids A-G; 0–64 μg/mL) and the mean IC_50_ and minimal inhibitory concentrations (MIC) for each peptide was determined. Growth was monitored spectrophotometrically and cell density (O. D. 600 nm) was determined after 96 h. Each bacterium was also grown anaerobically in the presence or absence of the antimicrobial peptoid exhibiting most efficacious anti-microbial activity (lowest mean IC_50_ concentrations) and CBD (5 μg/mL). Subsequently, multiple additional species of oral spirochetes (*Treponema putidum 700334, Treponema vincentii 700013, Treponema medium 700293*) and strains of *P. gingivalis* (W83 and four low-passage clinical isolates, 4162, 5607,8012, 10802c) were tested for sensitivity to peptoids E and B, respectively, based on the generated *T. denticola* ATCC 35405- and *P. gingivalis* ATCC 33277-specific data. The mean IC_50_ and minimal inhibitory concentrations (MIC) for the peptoids and bacteria of interest were determined. All peptoids were supplied by Maxwell Biosciences, Inc. (Austin, TX).

### Transmission electron microscopy

*Porphyromonas gingivalis* ATCC 33277 and *T. denticola* ATCC 35405 were exposed, or not, to CBD (0.2 and 5 μg/mL, respectively) or to peptoid B or E (mean IC_50_ concentrations, Table 1), respectively, and ultrastructural characteristics examined by electron microscopy. Briefly, *P. gingivalis* and *T. denticola* cells were harvested from triplicate cultures at 16 h (100 x ***g***, 30 min) and 65 h (900 x ***g***, 4 min), respectively. Pelleted cells were stored in fixative (2% glutaraldehyde in 0.1 M sodium cacodylate buffer, pH 7.4). Subsequently, all samples were prepared in 1% low temperature agarose prior to processing in a Leica Tissue processor. Samples were washed three times for 10 min with 0.1 M sodium cacodylate buffer followed by staining with 1% osmium tetroxide, 1 h. The samples were rinsed for an additional 10 min in 0.1 M sodium cacodylate buffer and stained en bloc with 1% uranyl acetate, 30 min. Samples were rinsed twice with distilled water then dehydrated progressively with 30, 50, 70, 90 and 95% ethanol, 10 min, and three times with anhydrous ethanol, 15 min. Embedding was performed with 1:1 Spurr’s resin and anhydrous ethanol with an increased concentration of 3:1 resin and anhydrous ethanol for one hr. Samples were placed in 100% resin under vacuum for 24 h without agitation then cured at 70 °C for 24 h. The embedded samples were sectioned to 100 nm thickness with a Lecia UC7 ultramicrotome and placed onto nickel grids with formvar support film. Post lead citrate staining was performed for 2 min, followed by rinsing with DI water. Samples were imaged with a Hitachi HT-7700 TEM at 80 kV. Imaging was performed using the facilities at the University of Louisville Micro/Nano Technology Center.

**Table 1:** Inhibition [IC_50_, (MIC)] of oral pathobionts by antimicrobial peptoids.

Peptoid	A	B	D	E	F
*T. denticola*	19, (32)	14, (32)	22, (32)	18, (32)	16, (32)
*P. gingivalis*	26, (32)	26, (32)	26, (32)	12, (16)	27, (32)

*T. denticola* ATCC 35405 and *P. gingivalis* ATCC 33277 were grown in the presence or absence of a panel of cathelicidin mimicking potential antimicrobial peptides. I. C._50_ and MIC (μg/ml)) values are presented.

## Results

### Cannabis use is associated with an oral dysbiosis *in vivo*

**Figure 1:**
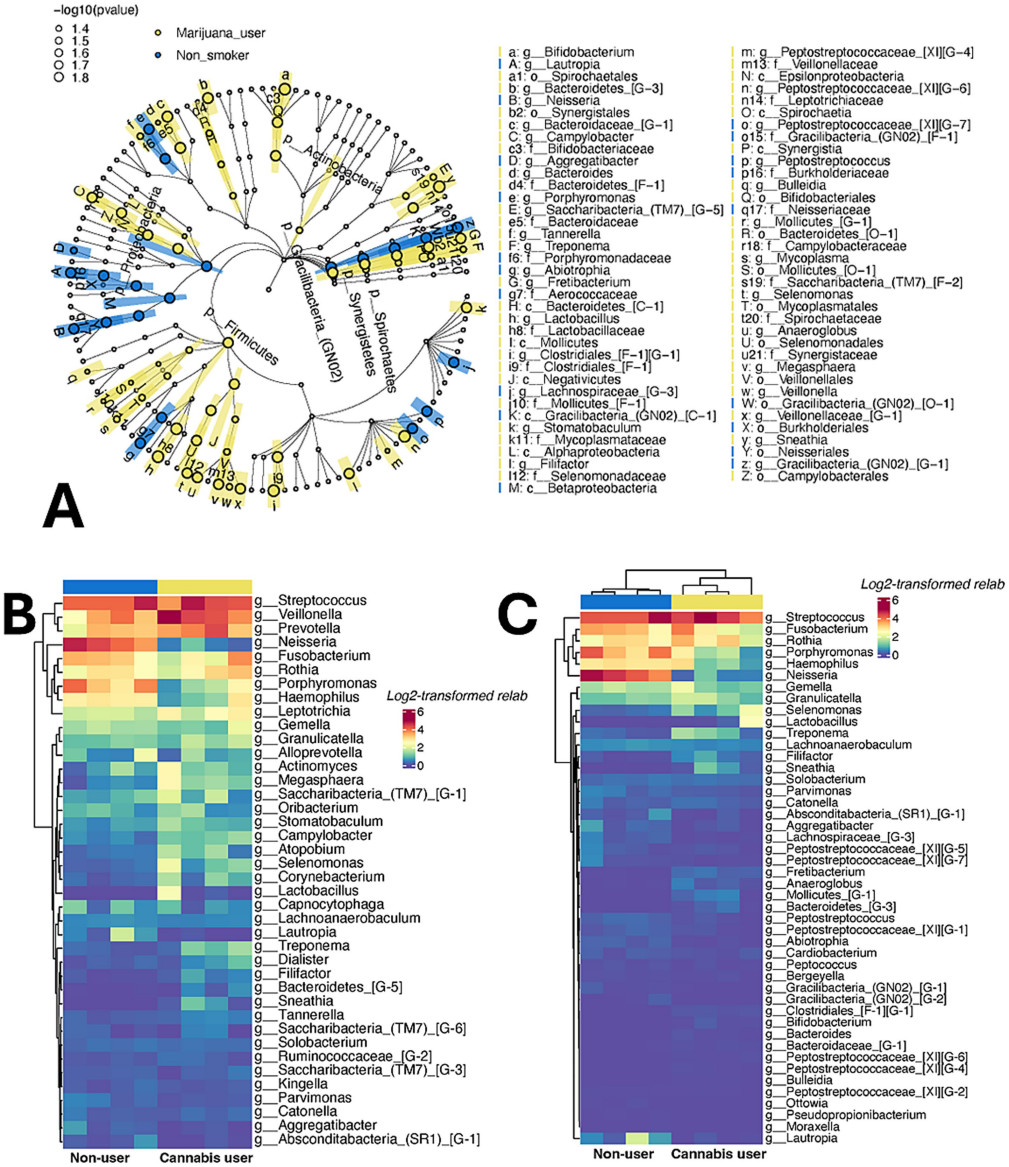
Specific microbial signatures differentiate cannabis users and non-users. **(A)** LEfSe cladogram of differentially enriched bacterial taxa in cannabis users (yellow traces) and non-users (blue traces). **(B)** Hierarchically row-clustered heatmap of the top 40 most relatively abundant genera (log_2_ transformed relative abundance). **(C)** Hierarchically row- and column-clustered heatmap of bacterial genera that were significantly enriched or depleted between cannabis users and non-users identified by ANCOM-BC (log_2_ transformed relative abundance). Each row represents a bacterial genus, and each column corresponds to an individual participant. The color bar above the heatmap indicates groups the samples belong to. All subjects had periodontitis and were non-tobacco users as described in Xu et al. (2021).

We examined the salivary microbiome of cannabis users and non-users with periodontitis who did not use tobacco products utilizing a subset of subjects from a prior clinical study (Xu et al., 2021). Cannabis use appears to be associated with a profound, differentiative oral dysbiosis as determined by PCoA plots based on Weighted Unifrac distances (Supplementary Figure 1A); and Cytoscape networking (Supplementary Figure 1B). LefSe cladogram analysis of differentially enriched bacterial taxa (Figure 1A); and heat mapping of hierarchically clustered genera (Figures 1B,C) provided information on the more prominent species in each group. Species representative of those bacterial genera that differentiated cannabis users in the *in vivo* convenience sample were selected for further *in vitro* investigations of sensitvity and resistance to antimicrobial phytocannabinoids. Those genera enhanced in cannabis users included *Campylobacter*, *Corynebacterum*, *Haemophilus*, *Treponema* and *Veillonella*. Those genera more prevalant in non-users included *Aggregatibacter*, *Fusobacterium*, *Porphyromonas* and *Tannerella*. *The complete data set supporting the microbiome findings of this study is openly available in GEO at* *https://www.ncbi.nlm.nih.gov/bioproject/PRJNA779977/*.

### Differential cannabinoid sensitivity and resistance profiles among oral bacteria *in vitro*

Next, the sensitivities to CBD of multiple oral species representative of genera suggested to be enriched or depleted in the oral cavity, in the context of cannabis use, were determined, as presented in Tables 2, 3. Strikingly, and despite low numbers in the human-derived data set, *in vivo* microbial profiling of CBD sensitivity essentially predicted laboratory sensitivity and resistance among oral bacteria. The exception to the rule was *Tannerella*, with *T. forsythia* being predicted to be cannabinoid-resistant *in vivo* but found to be cannabinoid-sensitive in a laboratory setting.

**Table 2:** CBD-sensitive oral bacteria.

Bacterium	*In vivo* CBD prediction*	*In vitro* CBD Phenotype	Lab MIC
*Aggregatibacter actinomycetemcomitans*	SENSITIVE	SENSITIVE	1.56 μg/mL
*Fusobacterium nucleatum*	SENSITIVE	SENSITIVE	1 μg/mL
*Porphyromonas gingivalis*	SENSITIVE	SENSITIVE	5 μg/ml
*Streptococcus gordonii*	–	SENSITIVE	0.1 μg/ml
*Streptococcus mutans*	–	SENSITIVE	0.1 μg/ml
*Tannerella forsythia*	RESISTANT	SENSITIVE	5–10 μg/mL

A single species representative of bacterial genera shown to be under-represented in cannabis users was tested for sensitivity to CBD *in vitro*. *S. gordonii* and *S. mutans* were also examined. *Sensitivity to CBD predicted by heat map and/or LDA analysis of the *in vivo* microbiome in users and non-users (Figure 1).

**Table 3:** CBD-resistant oral bacteria.

Bacterium	*In vivo* CBD prediction*	*In vitro* CBD Phenotype
*Campylobacter gracilis*	RESISTANT	RESISTANT
*Corynebacterium durum*	RESISTANT	RESISTANT
*Haemophilus parainfluenzae*	RESISTANT	RESISTANT
*Treponema denticola*	RESISTANT	RESISTANT
*Treponema putidum*	RESISTANT	RESISTANT
*Veillonella parvula*	RESISTANT	RESISTANT

A single species representative of bacterial genera shown to be over-represented in cannabis users (Figure 1) was tested for sensitivity to supraphysiological doses of CBD *in vitro*. Sensitivities were examined at hyperphysiological CBD doses of up to 1,000 μg/mL. *Sensitivity to CBD predicted by heat map and/or LDA analysis of the *in vivo* microbiome in users and non-users (Figure 1).

### Multiple strains of *Porphyromonas gingivalis* are sensitive to CBD

**Figure 2:**
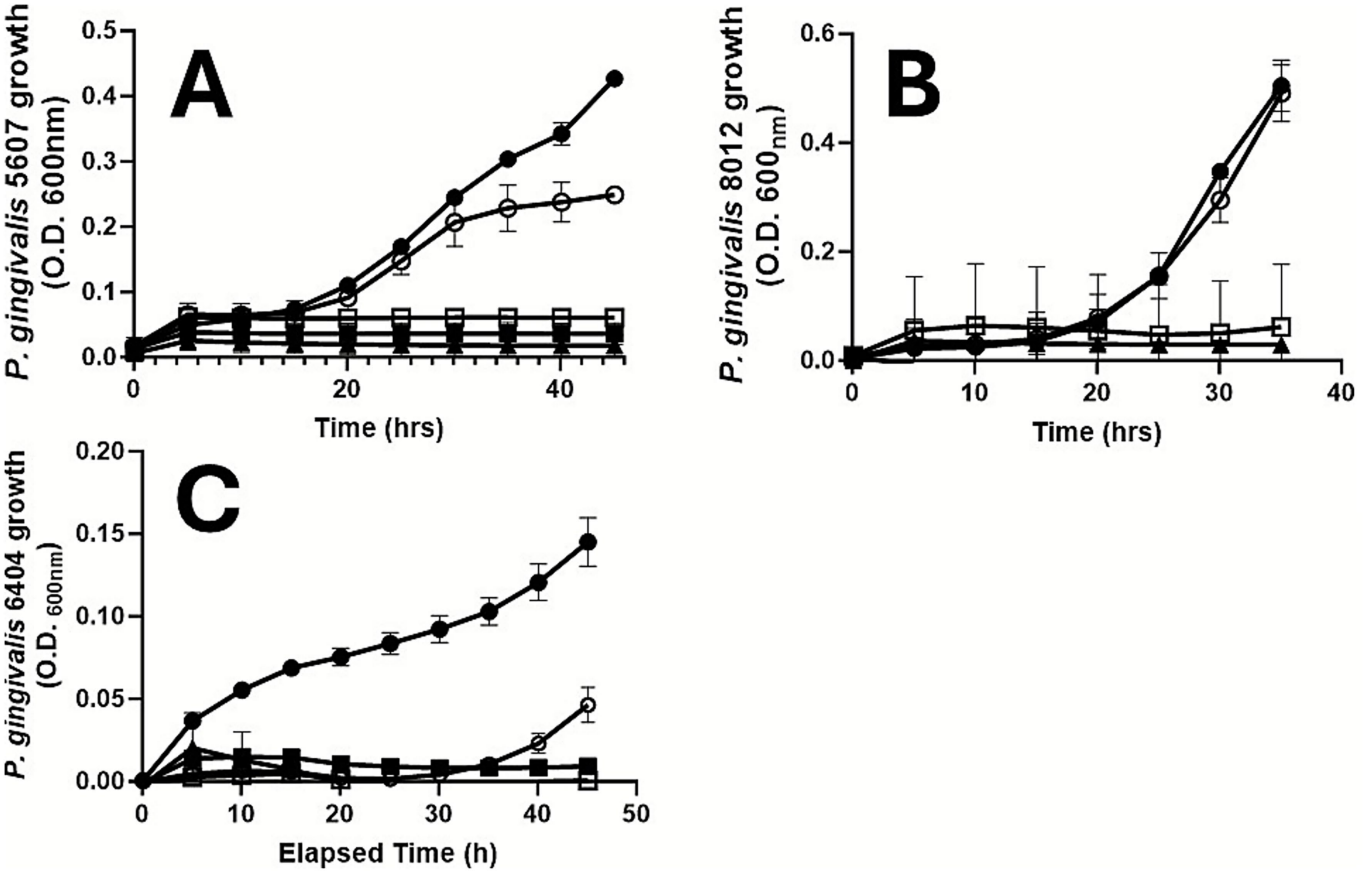
Multiple *P. gingivalis* strains are sensitive to CBD. Growth of *P. gingivalis* **(A)** 5607, **(B)** 8012 and **(C)** 6404 was monitored spectrophotometrically in the presence or absence of CBD. ●, ○, ■, □, ▲ represent 0.0 (vehicle), 0.1, 1.0, 5.0, and 10.0 ug/ml CBD, respectively. Data represent mean (S.D.) values. We have previously reported that the *P. gingivalis* type strain, ATCC 33277, is also CBD-sensitive (Gu et al., 2019).

We have previously shown that multiple strains of *T. denticola* (ATCC 35404, 35405, 33520 and 33521) as well as *Treponema putidum* ATCC 700334 are resistant to CBD in an *in vitro* setting (Tan et al., 2024). We have also reported that growth of the type strain of *P. gingivalis* (ATCC 33277) is abrogated upon phytocannabinoid exposure (Gu et al., 2019). We now establish that multiple other low-passage clinical isolates of this bacterium (*P. gingivalis* 5607, 8012, 6404) are similarly sensitive to CBD (Figure 2).

### Select antimicrobial peptoids exhibit antimicrobial activity against *Porphyromonas gingivalis* and *Treponema denticola*

As shown in Table 1, both *P. gingivalis*, a tobacco-enhanced bacterium (Tan et al., 2024), and *T. denticola*, a potentially cannabis-enhanced bacterium (Gu et al., 2019; Tan et al., 2024), are highly susceptible to select antimicrobial peptoids, with peptoids E and B showing the highest efficacy against each species, respectively.

### Antimicrobial peptoids alter CBD toxicity toward *Treponema denticola*

**Figure 3:**
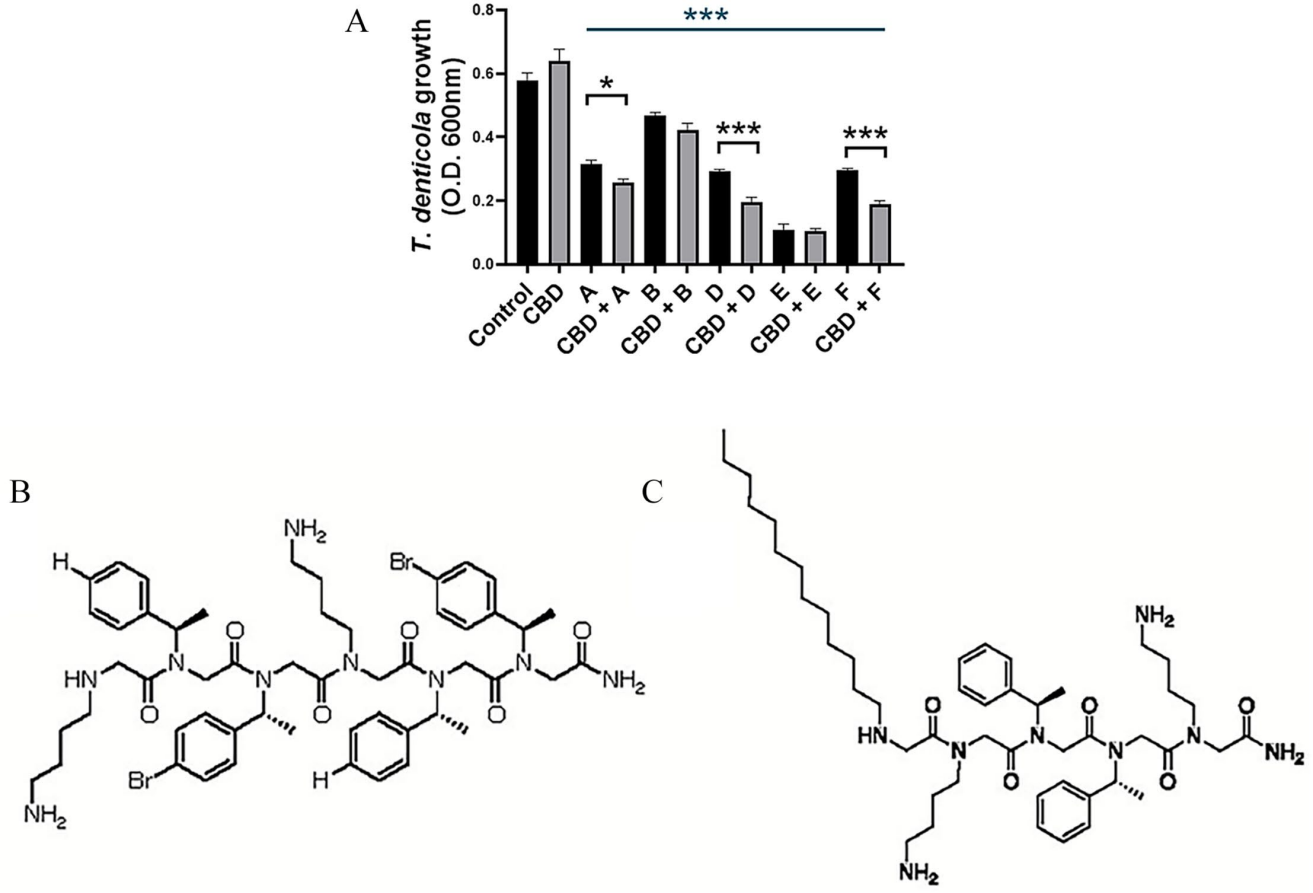
Synergistic antimicrobial activity of CBD and select antimicrobial peptoids against *T. denticola*. **(A)** *T. denticola* ATCC 35405 was grown anaerobically in the presence or absence of peptoids A, B, D, E, or F (at mean IC_50_ concentration; Table 1) and CBD (5 μg/mL). Growth was monitored spectrophotometrically and cell density (O. D. 600 nm) plotted after 96 h. Structure of cathelicidin-mimicking peptoid B **(B)** and peptoid E **(C)**, respectively. The structures of peptoid B (MXB-2) and peptoid E (MXB-5) were first described in Diamond et al. (2021).

Upon exposure to both anti-spirochetal peptoids (A, B, C, D, F) and CBD, further growth inhibition of *T. denticola* is noted compared to LL37-based peptoids alone, as shown in Figure 3A. The sequences of the most efficient antimicrobial peptoids, peptoid B [MXB-5; H-Ntridec-*N*Lys-*N*spe-*N*spe-*N*Lys-NH_2_] and peptoid E [MXB-2; H(-*N*lys-*N*spe-*N*spe)_4_-NH_2_] are presented in Figures 3B,C, respectively.

### Select antimicrobial peptoids exhibit antimicrobial activity against multiple *Treponema* species and *Porphyromonas gingivalis* strains

As shown in Table 4, multiple oral spirochete species and strains and several *P. gingivalis* strains are highly susceptible to antimicrobial peptoids E and B, respectively.

**Table 4:** Inhibition of oral pathobionts by peptoid B (oral spirochete species) and peptoid E (*P. gingivalis* strains).

Susceptibility	*T. putidum*	*T. vincentii*	*T. medium*	W83	5607	8012	10802c	4612
MIC (μg/ml)	32	64	16	16	16	8	16	16
I. C._50_ (μg/ml)	28	15	13	10	9	7	9	9

Multiple oral Treponema species and *P. gingivalis* strains were grown in the presence or absence of peptoid E and B, respectively. MIC and I. C._50_ (μg/ml) values are presented for each bacterium of interest.

### CBD and antimicrobial peptoids alter *Treponema denticola* and *Porphyromonas gingivalis* ultrastructure

**Figure 4:**
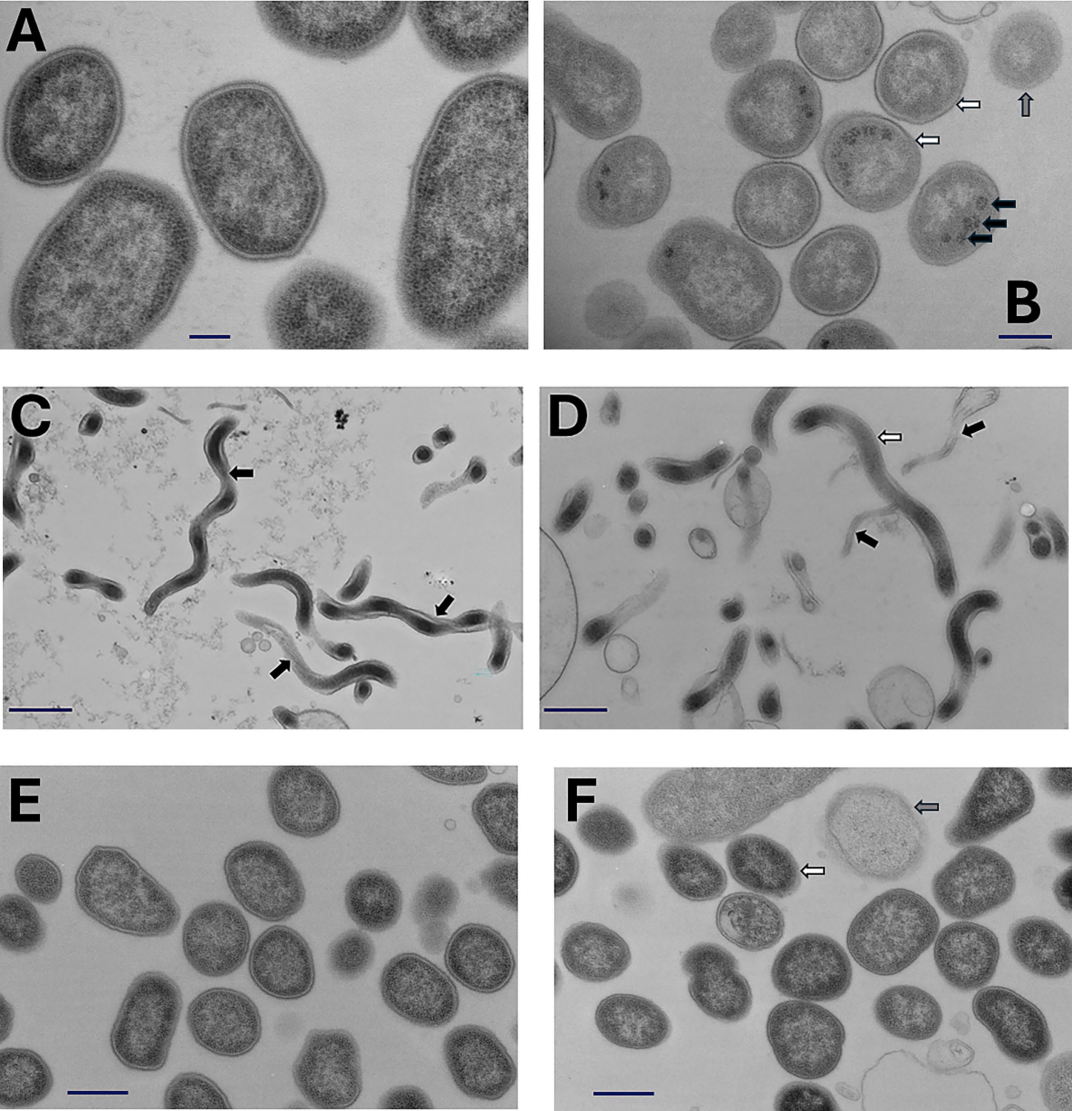
CBD and antimicrobial peptoids induce ultrastructural abnormalities in sensitive oral bacteria. CBD, which is toxic to *P. gingivalis*, induced electron dense granular structures (black arrows) within the cytoplasm of this bacterium **(A)** [control] and **(B)** [CBD, 0.2 μg/mL]. CBD-treated cells exhibited less well-defined cell wall ultrastructure than their control counterparts (white bars) and were smaller. The presence of ghost cells was also noted (gray bars). Peptoid B treatment (14 μg/mL) induced clear morphological changes in *T. denticola*. Control cells **(C)** appeared intact with clear periplasmic flagella (black arrows), whereas peptoid-exposed *T. denticola* cells **(D)** appeared more translucent (white arrow), with exposed flagella (black arrows), indicative of a compromised outer membrane structure. Peptoid E-treated *P. gingivalis* 12 μg/mL **(F)** exhibited a more diffuse surface (white arrows) than untreated control cells **(E)** with ghost cells of this Gram-negative pathobiont also visualized (gray arrows). Peptoid E-treated cells were also smaller. The scale bars on panels **A** and **B** represent 600 nm. The bars on panels **C**, **D**, and **F** represent 400 nm. The bar on panel **E** represents 200 nm.

CBD, which is toxic to *P. gingivalis*, induced electron dense granular structures within the bacterial cytoplasm of this bacterium (Figures 4A,B). Peptoid treatment induced distinct morphological changes in *T. denticola* (Figures 4C,D). Control cells appeared intact with clear periplasmic flagella, whereas peptoid-exposed *T. denticola* cells appeared more translucent, with exposed flagella, indicative of a compromised outer membrane structure. Further, increased ghost cells were apparent in the peptoid-treated spirochetes. Peptoid-treated *P. gingivalis* (Figures 4E,F) appeared smaller and exhibited a more diffuse surface than untreated control cells with ghost cells of this Gram-negative pathobiont also visualized.

## Discussion

Oral microbiome analyses, presented herein, suggest that cannabis use appears to be associated with a profound oral dysbiosis. Beta-diversity analysis using Weighted Unifrac distance, which considers the presence and absence, relative abundance, and phylogenetic relatedness of bacteria in two sample sets, combined with principal coordinate analysis, indicates a clear differentiation of the oral microbiome of cannabis users from non-users at high confidence. Such differentiation is also abundantly apparent using network analysis where amplicon sequence variants (sample nodes) distinctly connect to either users or non-users with few shared interactions. LefSe cladograms of differentially enriched bacterial taxa, in addition to heatmaps depicting hierarchically clustered genera based on the log_2_-transformed relative abundances (with and without ANCOM-BC strapping) show multiple genera that differentiate cannabis users (*Campylobacter*; *Corynebacterium*; *Haemophilus*; spirochetes including *Treponema*; *Veillonella*) and non-users (*Aggregatibacter*; *Fusobacterium*; *Porphyromonas*), many of which are readily cultivable.

We are aware that the sample size for such *in vivo* analyses is small and, thus, larger studies are warranted to establish cannabis-associated microbial fluxes in the mouth with a higher degree of certainty. Nevertheless, laboratory profiles of CBD resistance and susceptibility among readily cultured oral bacteria were, essentially, as predicted by the *in vivo* data set.

*Campylobacter gracilis*, is a Gram-negative anaerobic rod, considered a periodontitis-associated pathobiont (Haririan et al., 2014), particularly in obese and/or diabetic individuals (Alarcon-Sanchez, 2024; Rodriguez-Hernandez et al., 2019). *Corynebacterium durum*, a rarely studied Gram-positive bacterium, is likely to be a genuine commensal, considering the available microbiome data, its inability to illicit a meaningful response from innate sensor cells of the periodontium, and promotion of health-associated streptococcal growth (Redanz et al., 2021; Treerat et al., 2020). *Haemophilus parainfluenzae* has been traditionally considered a commensal in the oral cavity. However, recent data suggest disease association, including during clinical inflammation (Park et al., 2015), while it has clear pro-inflammatory, pathogenic potential in the GI tract (Sohn et al., 2023; Kawamoto et al., 2021; Diao et al., 2021). Thus, evaluations of the pathogenicity of *H. parainfluenzae* are currently fluid. *Treponema denticola*, an unusual anaerobic Gram-negative pathobiont and best understood oral spirochete, has been reported to dominate in deep gingival pockets (Byrne et al., 2022; Jepsen et al., 2021), persist in recalcitrant sites (Byrne et al., 2022), and its presence has been correlated with increased inflammation, as monitored by the gingival bleeding response (Bertelsen et al., 2022). We have previously reported that *Treponema putidum* and multiple strains of *T. denticola* are phytocannabinoid resistant (Tan et al., 2024). *Veillonella parvula*, a Gram-negative anaerobic coccus, is generally considered a bridging organism facilitating the development of mature biofilms of high diversity containing multiple pathobionts (Sakanaka et al., 2022; Dorison et al., 2024). We show that all these organisms are predicted to be cannabinoid-resistant by the *in vivo* microbiome data and are, indeed, confirmed as such in the laboratory setting. Further, and in keeping with our own data, *Veillonella* and *Corynebacterium* have recently been suggested to be more prevalent in the saliva of cannabis users compared to non-users (Panee et al., 2024). Thus, promotion of cannabinoid-resistant bacteria in the oral cavity may lead to a dysbiosis that may prime for increased susceptibility to destructive periodontal disease.

With the exception of *T. forsythia* which was predicted to be CBD-resistant, those bacteria associated with non-cannabis use status and examined *in vitro* were shown to be cannabinoid sensitive. *Aggregatibacter actinomycetemcomitans*, a Gram-negative facultative anaerobe, is best known as a Grade C molar/incisor pattern periodontitis pathobiont (Alamri et al., 2023). *Fusobacterium nucleatum*, a Gram-negative, anaerobe, is known to co-aggregate with multiple other plaque bacteria, so assisting in the maturation of dental plaque (Chen et al., 2022). *Porphyromonas gingivalis*, the best studied oral pathobiont, is a highly proteolytic Gram-negative anaerobe that is particularly well-adapted to the oral cavity of tobacco smokers (Tan et al., 2024). We have previously shown that the *P. gingivalis* type strain, ATCC 33277, is CBD-, CBN- and THC-sensitive (Gu et al., 2019). Herein, we have additionally found that several low passage *P. gingivalis* isolates (5607, 8012, 6404) are phytocannabinoid sensitive. Thus, some key periodontal pathogens may be underrepresented in cannabis users, suggesting that the microbial signals underlying periodontitis in cannabis users may differ from other groups of individuals studied previously.

In support of our findings, Newman et al. have previously observed profound cannabis-induced microbial shifts on healthy oral mucosa (tongue and larynx) that included a suppression of both *P. gingivalis* and *F. nucleatum* (Newman et al., 2019). While ours is the first study to attempt to relate *in vivo* and *in vitro* cannabis-related resistance traits, prior studies have examined oral bacteria for phytocannabinoid susceptibility in a non-systematic manner. Santos et al. (2025) have confirmed our initial report of *P. gingivalis* laboratory sensitivity to cannabinoids (Gu et al., 2019) as well as the sensitivity of *F. nucleatum* and *A. actinomycetemcomitans*. Other oral bacteria reported to be CBD-sensitive include *Actinomyces naeslundii* (Santos et al., 2025); and *Peptostreptococcus anaerobius* (Santos et al., 2025) and related spp (Lakes et al., 2024). However, heat mapping of the top 40 differentiating genera in the present study suggest the overall burden of *Actinomyces* spp. to be higher in cannabis users.

We also examined the laboratory cannabinoid sensitivity profiles of *S. mutans* and *S. gordonii*. *S. mutans*, a primary agent of dental caries, has previously been reported to be sensitive to cannabigerol (CBG) and CBD (Garzon et al., 2024; Avraham et al., 2023; Barak et al., 2022; Aqawi et al., 2021). Our data concurs with these findings. *S. gordonii* is a key partner organism of *P. gingivalis* and is considered an accessory pathobiont (Bagaitkar et al., 2011; Park et al., 2005; Kuboniwa et al., 2017). We show that *S. gordonii* is highly sensitive to CBD. Therefore, cannabinoids may inhibit *P. gingivalis* colonization due to both direct toxicity and to abrogation of an important accessory pathobiont. The cannabinoid-related suppression of *Porphyromonas gingivalis*, a classic Gram-negative, anaerobic periodontal pathobiont, is particularly fascinating.

Concomitantly, there is an expanding interest in the use of cannabinoids as antimicrobial agents to combat increasing antibiotic resistance among multiple important pathogens. Antimicrobial efficacy of cannabinoids has been suggested for cannabinoids against, for example, *Escherichia coli*, *Staphylococcus aureus,* and *Pseudomonas aeruginosa,* as recently reviewed (Hong et al., 2022). A potentially important counterpoint to this is that cannabinoid-based therapeutics could provide a competitive advantage to cannabinoid-resistant bacteria, a phenomenon perhaps particularly prescient in diseases associated with complex microbial communities, such as periodontitis.

Antimicrobial peptides are important components of the innate immune defense system. However, AMP-based peptide therapeutics are problematic as they are expensive to manufacture and, more importantly, are highly susceptible to peptidolytic degradation *in vivo*. To this end, inexpensive, and proteolytically stable mimics of the peptides have been developed, including short oligomeric mimetics and peptoids (Diamond et al., 2021; Tew et al., 2010; Chongsiriwatana et al., 2008). We and others have previously shown that select LL-37 mimicking peptoids are effective against multiple and varied enveloped viruses, including HSV-1, SARSCov2, Zika, Rift Valley Fever, and chikungunya (Diamond et al., 2021; Tate et al., 2023) and against specific bacteria, *Pseudomonas aeruginosa*, a Gram-negative, and *Staphylococcus aureus*, a Gram-positive bacterium (Lin et al., 2022). Herein, we show for the first time that several antimicrobial peptide-informed peptoids are efficacious growth inhibitors of both a model tobacco-enriched pathobiont, *P. gingivalis*, and a model cannabinoid-resistant bacterium, *T. denticola*. Treatment of *P. gingivalis* ATCC 33277 with the I.C._50_ of most effective peptoid tested, (peptoid E, 12 μg/mL), was associated with bacteria that appeared smaller and exhibited a more diffuse surface architecture than untreated control cells. Ghost cells of this Gram-negative pathobiont were also visualized. Peptoid treatment (peptoid B, 14 μg/mL) also induced clear morphological changes in *T. denticola*. Control spirochetes appeared intact with visible periplasmic flagella whereas peptoid-exposed *T. denticola* cells appeared more translucent, with exposed flagella, indicative of a compromised outer membrane structure. Peptoid B-treated cells were also smaller. While the structural determinants that lead to the different activities of the most active peptoids against the two different species of bacteria are unclear, the direct membrane activity of these compounds appears to be related to their cationic nature, which has been shown to directly interact with phosphatidylserine headgroups on the outer leaflet of the microbial membrane. Further, their hydrophobic nature leads to membrane disruption (Tate et al., 2023). We also report that the antimicrobial activities of these cathelicidin-based peptoids extends beyond the type strains of *P. gingivalis* and *T. denticola*, with efficacy demonstrated against multiple addition *P. gingivalis* strains and several oral anaerobic spirochete species. Presumably due to peptoid-induced outer membrane instability, peptoid treatment also rendered CBD toxic to the normally resistant spirochete, *T. denticola*.

While the *in vivo* data are reflective of biofilm formation *in situ* and largely concur with our laboratory profiling, it is important to note that our *in vitro* sensitivity assays have used planktonic cells. Examinations of the influence of CBD and peptoid mimetics on mono- and multispecies biofilms would, therefore, better reflect potential *in vivo* antimicrobial efficacy.

The mechanisms of microbicidal action of phytocannabinoids are, perhaps surprisingly, not fully understood. Various possibilities, however, have been proposed, including induction of membrane instability, dysregulation of cell division mediators and suppression of protein and peptidoglycan synthesis (Hong et al., 2022). We selected *P. gingivalis* as a model CBD-sensitive oral bacterium for transmission electron microscope observations. Interestingly, CBD exposure led to obvious ultrastructural differences relative to untreated cells. Dark granular structures were induced and, while their relevance is not defined, similar structures have been reported in bactericidal polyphosphate- and trans-cinnamaldehyde-treated *P. gingivalis* cells (Moon et al., 2011; Gan et al., 2023). CBD-treated *P. gingivalis* cells also appeared smaller and with less distinct cell wall ultrastructure than their control counterparts.

In summary, we show that *in vivo* profiling of the oral microbiome in cannabis users and non-users suggests that specific enriched and depleted bacterial signatures differentiate these groups and reflect sensitivity versus resistance to phytocannabinoids in the laboratory. Larger studies will be required to confirm this phenomenon. Further, both CBD and AMP-based peptoids induce substructural features in susceptible oral bacteria that are at odds with healthy control cells. Future studies that more definitively define cannabis-induced microbial flux in the oral cavity are warranted. It may also be worthwhile to further explore antimicrobial peptoids as potential periodontal therapeutics whose value may be greatest in environmentally vulnerable populations, tobacco and cannabis users in particular. Finally, we should be cognizant that the complex microbial interactions known to occur in oral biofilms promote genetic exchanges that may have the potential to facilitate the exchange of cannabinoid-resistant traits.

## Data Availability

The sequencing data is available at: https://www.ncbi.nlm.nih.gov/bioproject/PRJNA779977/.

## Supplementary material

Supplementary Figure 1 The oral microbiome of cannabis users is distinct from non-users. **(A)** PCoA plots based on Weighted Unifrac distances establish a distinct microbiome in cannabis users and non-users. Each symbol represents a sample from cannabis users (yellow) or non-users (blue). Clusters were determined by PERMANOVA and the ellipses indicate 95% CI. **(B)** Cytoscape network illustrating sample–amplicon sequence variant (ASV) interaction among the cannabis users (yellow squares) and non-users (blue squares), with ASVs presented as white circles. The network highlights group-specific microbial patterns, where samples with a large degree of ASV overlap (weighed by abundance) are clustered close to each other. Edges represent the interaction between sample and ASV and are colored based on sample type. The layout was generated using Cytoscape’s Edge-Weighted Spring Embedded algorithm, which emphasizes connectivity strength. All subjects had periodontitis and were non-tobacco users as described in Xu et al. (2021).
