# Supplementary figures and images for: Identification of cannabinoid-sensitive and -resistant oral bacteria
Source: Front Microbiol. 2026 Jan 5;16:1709243. doi: 10.3389/fmicb.2025.1709243 (PMC12812963; doi:10.3389/fmicb.2025.1709243)

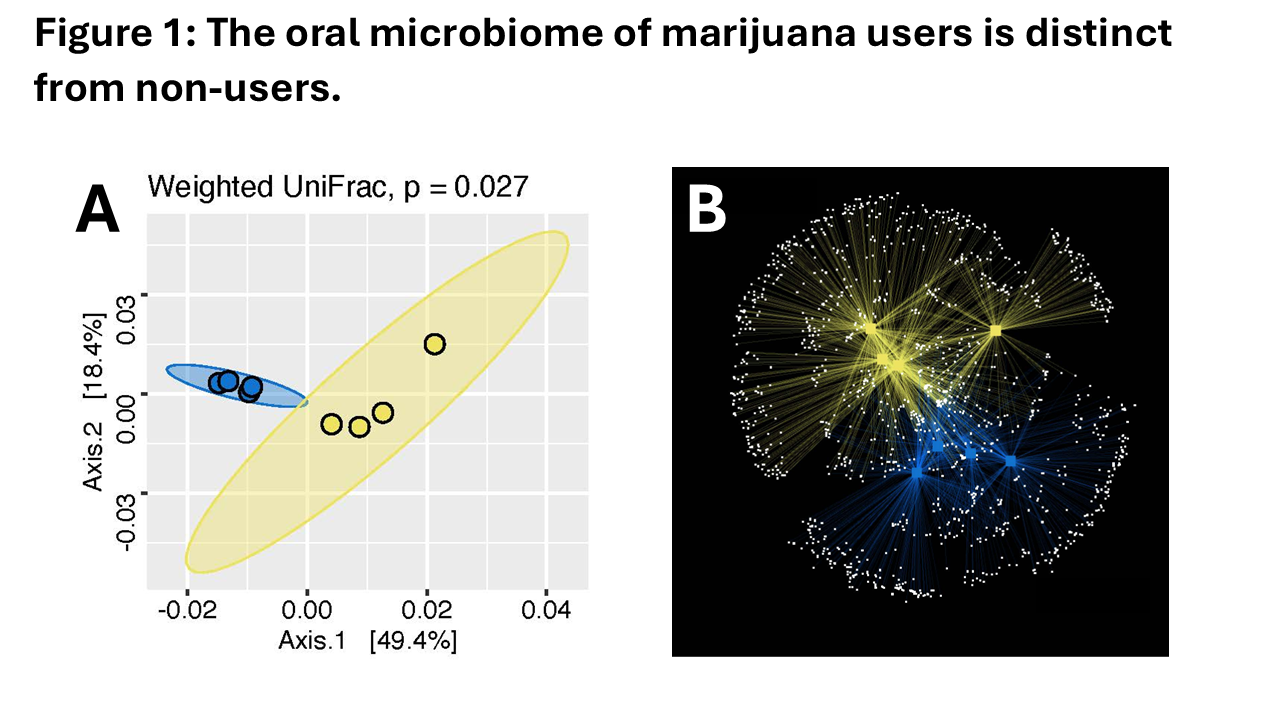

Supplement: Supplementary Figure 1 — The oral microbiome of cannabis users is distinct from non-users. (A) PCoA plots based on Weighted Unifrac distances establish a distinct microbiome in cannabis users and non-users. Each symbol represents a sample from cannabis users (yellow) or non-users (blue). Clusters were determined by PERMANOVA and the ellipses indicate 95% CI. (B) Cytoscape network illustrating sample–amplicon sequence variant (ASV) interaction among the cannabis users (yellow squares) and non-users (blue squares), with ASVs presented as white circles. The network highlights group-specific microbial patterns, where samples with a large degree of ASV overlap (weighed by abundance) are clustered close to each other. Edges represent the interaction between sample and ASV and are colored based on sample type. The layout was generated using Cytoscape’s Edge-Weighted Spring Embedded algorithm, which emphasizes connectivity strength. All subjects had periodontitis and were non-tobacco users as described in Xu et al. (2021). [file Image_1.tif]
