# Supplementary material for: Identification of cannabinoid-sensitive and -resistant oral bacteria
Source: Front Microbiol. 2026 Jan 5;16:1709243. doi: 10.3389/fmicb.2025.1709243 (PMC12812963; doi:10.3389/fmicb.2025.1709243)
Supplement: Supplementary file 2 [file Presentation_1.pptx]

## Slide 1
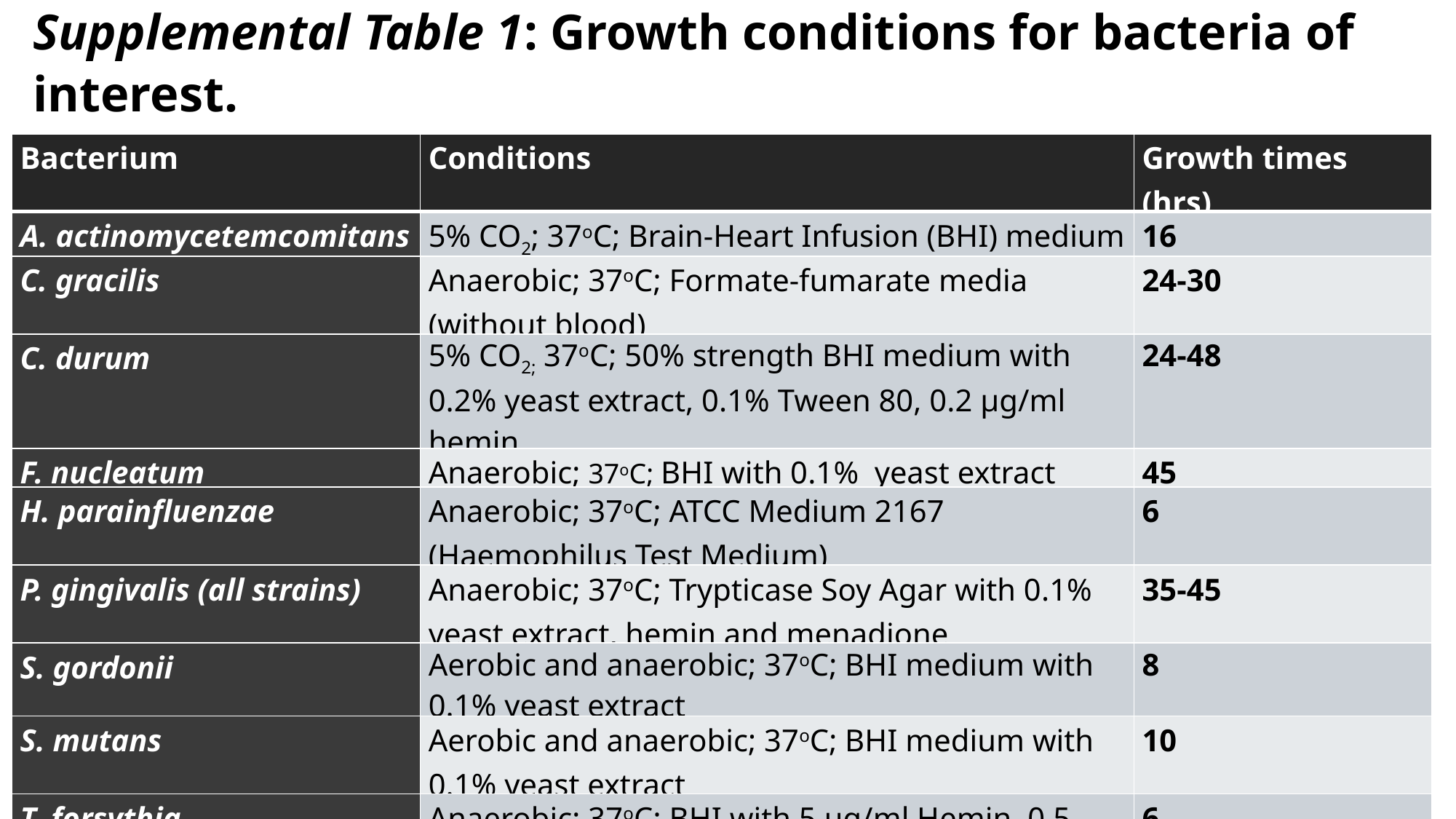

Supplemental Table 1: Growth conditions for bacteria of interest.
| Bacterium | Conditions | Growth times (hrs) |
| --- | --- | --- |
| A. actinomycetemcomitans | 5% CO2; 37oC; Brain-Heart Infusion (BHI) medium | 16 |
| C. gracilis | Anaerobic; 37oC; Formate-fumarate media (without blood) | 24-30 |
| C. durum | 5% CO2; 37oC; 50% strength BHI medium with 0.2% yeast extract, 0.1% Tween 80, 0.2 µg/ml hemin | 24-48 |
| F. nucleatum | Anaerobic; 37oC; BHI with 0.1%  yeast extract | 45 |
| H. parainfluenzae | Anaerobic; 37oC; ATCC Medium 2167 (Haemophilus Test Medium) | 6 |
| P. gingivalis (all strains) | Anaerobic; 37oC; Trypticase Soy Agar with 0.1% yeast extract, hemin and menadione | 35-45 |
| S. gordonii | Aerobic and anaerobic; 37oC; BHI medium with 0.1% yeast extract | 8 |
| S. mutans | Aerobic and anaerobic; 37oC; BHI medium with 0.1% yeast extract | 10 |
| T. forsythia | Anaerobic; 37oC; BHI with 5 µg/ml Hemin, 0.5 µg/ml menadione, 0.001% N-acetylmuramic acid and 5% bovine serum | 6 |
| T. denticola | Anaerobic; 37oC; Tryptone, yeast extract, gelatin, volatile fatty acids, and serum (TYGVS) medium | 100 |
| T. putidum | Anaerobic; 37oC; TYGVS | 100 |
| V. parvula | Anaerobic; 37oC; Todd Hewitt medium with 0.3% wt/vol yeast extract and 0.6% sodium lactate | 15 |
